# Human–machine partnership with artificial intelligence for chest radiograph diagnosis

**DOI:** 10.1038/s41746-019-0189-7

**Published:** 2019-11-18

**Authors:** Bhavik N. Patel, Louis Rosenberg, Gregg Willcox, David Baltaxe, Mimi Lyons, Jeremy Irvin, Pranav Rajpurkar, Timothy Amrhein, Rajan Gupta, Safwan Halabi, Curtis Langlotz, Edward Lo, Joseph Mammarappallil, A. J. Mariano, Geoffrey Riley, Jayne Seekins, Luyao Shen, Evan Zucker, Matthew P. Lungren

**Affiliations:** 1 Department of Radiology, Stanford University School of Medicine, 300 Pasteur Dr., H1307, Stanford, CA 94305 USA; 2 Unanimous AI, 2443 Fillmore Street #116, San Francisco, CA 94115-1814 USA; 3 Department of Computer Science, Stanford University School of Medicine, 353 Serra Mall (Gates Building), Stanford, CA 94305 USA; 4 Department of Radiology, Duke University Medical Center, Box 3808 Erwin Rd, Durham, NC 27710 USA

**Keywords:** Radiography, Computer science

## Abstract

Human-in-the-loop (HITL) AI may enable an ideal symbiosis of human experts and AI models, harnessing the advantages of both while at the same time overcoming their respective limitations. The purpose of this study was to investigate a novel collective intelligence technology designed to amplify the diagnostic accuracy of networked human groups by forming real-time systems modeled on biological swarms. Using small groups of radiologists, the swarm-based technology was applied to the diagnosis of pneumonia on chest radiographs and compared against human experts alone, as well as two state-of-the-art deep learning AI models. Our work demonstrates that both the swarm-based technology and deep-learning technology achieved superior diagnostic accuracy than the human experts alone. Our work further demonstrates that when used in combination, the swarm-based technology and deep-learning technology outperformed either method alone. The superior diagnostic accuracy of the combined HITL AI solution compared to radiologists and AI alone has broad implications for the surging clinical AI deployment and implementation strategies in future practice.

## Introduction

Recent notable applications of deep learning in medicine include automated detection of diabetic retinopathy, classification of skin cancers, and detection of metastatic lymphadenopathy in patients with breast cancer, all of which demonstrated expert level diagnostic accuracy.^1–3^ Recently, a deep-learning model was found to match or outperform human expert radiologists in diagnosing 10 or more pathologies on chest radiographs.^4,5^ The success of AI in diagnostic imaging has fueled a growing debate^6–9^ regarding the future role of radiologists in an era, where deep-learning models are capable of performing important diagnostic tasks autonomously and speculation surrounds whether the comprehensive diagnostic interpretive skillsets of radiologist can be replicated in algorithms. However, AI is also plagued with several disadvantages including biases due to limited training data, lack of cross-population generalizability, and inability of deep-learning models to contextualize.^8,10–12^

Human-in-the-loop (HITL) AI may offer advantages where both radiologists and machine-learning algorithms fall short.^13,14^ This paradigm allows leveraging all the advantages of AI models (i.e. rapid automated detection) but having a human at various checkpoints to fill gaps where algorithms are not confident in their probabilities or where they may fall short due to underlying biases. For example, a machine-learning algorithm could analyze a large dataset and provide output for the presence of disease in a short period of time, some with high confidence (i.e. high probability of the presence or absence of the disease relative to the probabilistic threshold for the detection of that disease) and others with low. The lower confidence outputs could then be validated by a human to create a combined better decision on the input; this approach could harness the best of human intelligence and artificial intelligence to create a collective super intelligence. Recent work has shown superior task performance of a combined human and AI augmented model compared to either human^15^ or machine alone.^15,16^ To date, however, no studies have harvested the full collective intelligence of a group of radiologists and then examined the performance of an augmented model.

In this study, we employ a novel collective intelligence platform called Swarm^17–19^ designed to amplify the accuracy of networked human groups by enabling the groups to work together in real-time systems modeled after biological swarms. In contrast to traditional crowds, swarm intelligence refers to stigmergic collaborative behavior of large groups of independent agents that form a closed-loop system, resulting in an collective super intelligence whose capacity exceeds that of any individual agent.^17,19^ The most studied form of swarm intelligence in nature is the honeybee swarm, which has been shown to make decisions through a process that is surprisingly similar to neurological brains.^20–22^ Both employ large populations of simple excitable units (i.e., bees and neurons) that work in unison to integrate noisy and incomplete information, weigh competing alternatives and converge on unified decisions in real-time synchrony. In both brains and swarms, outcomes are arrived at through a real-time competition among sub-populations of excitable units. When one sub-population exceeds threshold support, the corresponding alternative is chosen. In honeybees, this enables the colonies to converge on optimal decisions to highly complex problems, such as selecting an optimal home location from among a large set of alternatives.^21–23^

When using the platform with groups of radiologists, the swarm-based technology was applied to the diagnosis of pneumonia on chest radiographs. Diagnostic accuracy of the swarm-based technology was compared against the human experts alone and two state-of-the-art deep-learning AI models that have demonstrated expert level performance in automated pneumonia and multiple diagnosis detection, respectively.^5,6^ In addition, a novel combination of the swarm-based technology and the deep-learning AI models was compared against each of the methods in isolation.

## Results

### Experiment design

Complete details regarding the chest radiograph dataset, two deep-learning model architectures, and swarm-based collective intelligence platform is discussed in the online methods section. In brief, a total of 13 expert radiologists split over two sessions (7 radiologists in group A; 6 radiologists in group B) provided their estimate of the probability of the presence or absence of pneumonia on 50 chest radiographs, first alone then collectively using the real-time swarm platform. Two state-of-the art deep-learning models, CheXNet and CheXMax, were also used to evaluate the chest radiographs and the performance between individual human experts, real-time swarms, and AI were compared. Finally, a novel combination of the real-time swarm platform and the deep-learning models was compared against each method in isolation.

The aggregate performance of individual human experts was calculated in two ways: first, the average probability of all radiologists in each group was calculated and used as the crowd-based mean performance. Second, a crowd-based majority diagnosis was calculated using a vote—if more radiologists diagnosed pneumonia than no pneumonia using a 50% probability cutoff, the crowd-based majority diagnosis was pneumonia.

The probabilistic diagnoses from CheXNet and CheXMax were turned into binary classifications of pneumonia using a discrimination threshold—any probabilistic diagnosis above the threshold is assigned a prediction of “Pneumonia” and any diagnosis below the threshold is assigned a prediction of “No Pneumonia”. The thresholds for these algorithms were set to maximize the performance of the algorithms on their respective training data sets. The discrimination threshold for CheXNet is 50%, and the discrimination threshold for CheXMax is 4.008%.

### Diagnostic performance results

**Fig. 1:**
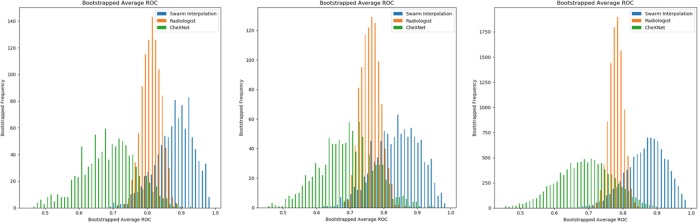
Bootstrapped average AUC curves. AUC curves show that the swarms (blue bars) outperform group A (left image), group B (middle image), and combined group (right image). Radiologists (orange bars) performances in diagnosing pneumonia. Swarm also outperforms CheXNet (green bars).

Results of the diagnostic performance of individuals, real-time swarms, and AI models are summarized in Tables 1 and 2. The individual radiologists outperformed CheXNet in diagnosing pneumonia in this study (AUC of 0.698 vs. 0.545, *p* < 0.01). CheXMax on the other hand, outperformed the individual radiologists (AUC of 0.938 vs. 0.698, *p* < 0.01). The swarm platform also outperformed the individual radiologists. For both swarm sessions, swarm interpolation achieved higher diagnostic accuracy than individual human performance, crowd-based performance, and CheXNet (Fig. 1). For group A, swarm achieved a statistically higher AUC of 0.840 [0.691, 0.937] compared to 0.763 [0.709, 0.817] (*p* < 0.05) average AUC of all radiologists in group A and to 0.685 [0.520, 0.854] (*p* < 0.01) AUC for CheXNet. For group B, the swarm had a statistically higher diagnostic accuracy than individual radiologists and CheXNet for all performance metrics (e.g. AUC of 0.889 vs. 0.810 and 0.685, respectively). When results from both swarm sessions were combined, swarm-based diagnoses resulted in statistically higher (*p* < 0.01) accuracies compared to radiologists and CheXNet (e.g. AUC of 0.868 vs. 0.785 and 0.685, respectively) (Fig. 1). There was no difference between CheXMax and the combined swarms in terms of accuracy or F1 score (accuracy of 82% vs. 84%, *p* = 0.423; F1 of 0.788 vs. 0.800, *p* = 0.34). CheXMax outperformed the combined swarm in AUC (0.938 vs. 0.868, *p* < 0.01), but the swarm outperformed CheXMax in terms of Brier score and mean absolute error (Brier scores of 0.287 vs. 0.134, *p* < 0.01; MAE of 0.357 vs. 0.233, *p* < 0.01).

**Table 1:** Diagnostic performance parameters for individual particpants, swarm sessions, and AI models.

Participants	Diagnostic performance parameters^a^				
	No. of correct (%)	Mean absolute error	Brier score	AUC	F1 score
Swarm sessions					
Group A (*N* = 7)					
Individual average	37 (75) [35, 40]	0.269 [0.231, 0.306]	0.188 [0.152, 0.274]	0.763* [0.709, 0.817]	0.687 [0.606, 0.736]
Crowd-based Majority	39 (78) [33, 44]	N/A	N/A	N/A	0.686 [0.533, 0.872]
Crowd-based mean probability	40 (80) [34, 45]	0.269 [0.198, 0.347]	0.145 [0.084, 0.213]	0.838 [0.686, 0.963]	0.722 [0.533, 0.872]
Swarm interpolation	42 (84) [37, 47]	0.235 [0.60, 0.324]	0.139 [0.070, 0.225]	0.840 [0.691, 0.937]	0.778 [0.588, 0.905]
Group B (*N* = 6)					
Individual average	39 (78)^¶^ [37,41]	0.260^¶^ [0.226, 0.295]	0.166^¶^ [0.135, 0.199]	0.814^¶^ [0.755, 0.870]	0.717^¶^ [0.626, 0.771]
Crowd-based majority	40 (80) [34, 45]	N/A	N/A	N/A	0.706 [0.483, 0.864]
Crowd-based mean probability	40 (80) [34, 45]	0.260^¶^ [0.189, 0.334]	0.135 [0.079, 0.208]	0.873 [0.730, 0.969]	0.706 [0.483, 0.864]
Swarm interpolation	42 (84) [37, 47]	0.231 [0.163, 0.314]	0.128 [0.069, 0.202]	0.883 [0.751, 0.964]	0.778 [0.600, 0.909]
Combined (*N* = 13)					
Individual average	38 (76)^†^ [36.5, 40]	0.266^†^ [0.240, 0.344]	0.179^†^ [0.154, 0.275]	0.785^†^ [0.740, 0.957]	0.698^†^ [0.635, 0.731]
Crowd-based majority	40 (80)^Ø^ [34, 45]	N/A	N/A	N/A	0.722^†^ [0.529, 0.867
Crowd-based mean probability	40 (80)^Ø^ [34, 45]	0.264^†^ [0.196, 0.344]	0.140 [0.083, 0.221]	0.853 [0.686, 0.960]	0.722^Ø^ [0.529, 0.867]
Swarm interpolation	84 (84) [78, 90]	0.233 [0.177, 0.279]	0.134 [0.096, 0.175]	0.868 [0.801, 0.933]	0.778 [0.685, 0.862]
Deep-learning models					
CheXNet	35 (70)^‡†^ [29, 41]	0.397^¶†^ [0.336, 0.461]	0.210^¶†^ [0.152, 0.274]	0.685*^¶†^ [0.520, 0.854]	0.545^¶†^ [0.333, 0.733]
CheXMax	41 (82) [35, 46]	0.357^†^ [0.249, 0.476]	0.287^†^ [0.184, 0.389]	0.938^†^ [0.864, 0.994]	0.800 [0.667, 0.917]
Augmented HITL model (combined swarm and CheXMax)	91 (91)^†?^ [86, 96]	0.356^†^ [0.297, 0.418]	0.287^†Ψ^ [0.211, 0.319]	N/A^b^	0.886^†?^ [0.819, 0.945]

*N/A* not applicable

*Indicates a statistically significant difference (*p* < 0.05) compared to group A swarm interpolation

^¶^Indicates a statistically significant difference (*p* < 0.01) compared to group B swarm interpolation

^‡^Indicates a statistically significant difference (*p* < 0.05) compared to group B swarm interpolation

^†^Indicates a statistically significant difference (*p* < 0.01) compared to combined swarm interpolation

^Ø^Indicates a statistically significant difference (*p* < 0.05) compared to combined swarm interpolation

^ϕ^Indicates a statistically significant difference (*p* < 001) compared to CheXMax

^Ψ^Indicates a statistically significant difference (*p* < 0.05) compared to CheXMax

^a^Data reported as mean [95% confidence interval] as applicable, unless otherwise specified

^b^AUC not applicable here as distribution of probabilities for swarm and CheXMax are centered about different averages (i.e. 50% vs. 4%, respectively)

**Table 2:** Sensitvity and specificity for individual particpants, swarm sessions, and AI models.

Participants	Diagnostic performance parameters^a^	
	Sensitivity	Specificity
Swarm sessions		
Group A (*N* = 7)		
Individual average	0.642 [0.579, 0.709]	0.819* [0.777, 0.862]
Crowd-based majority	0.650 [0.412, 0.783]	0.900 [0.800, 0.972]
Crowd-based mean probability	0.650 [0.462, 0.824]	0.900 [0.806, 1.00]
Swarm interpolation	0.700 [0.526, 0.875]	0.933 [0.852, 1.00]
Group B (*N* = 6)		
Individual average	0.633 [0.558, 0.704]	0.883 [0.845, 0.920]
Crowd-based majority	0.600 [0.421, 0.789]	0.933 [0.846, 1.00]
Crowd-based mean probability	0.600 [0.421, 0.789]	0.933 [0.846, 1.00]
Swarm interpolation	0.700 [0.500, 0.867]	0.933 [0.844, 1.00]
Combined (*N* = 13)		
Individual average	0.519^ϕ†^ [0.471, 0.568]	0.690 [0.654, 0.724]
Crowd-based majority	0.625^ϕØ^ [0.477, 0.721]	0.917 [0.857, 0.968]
Crowd-based mean probability	0.625^ϕ†^ [0.500, 0.744]	0.917^Ø^ [0.852, 0.968]
Swarm interpolation	0.700^Ψ^ [0.578, 0.814]	0.933^ϕ^ [0.855, 0.968]
Deep learning models		
CheXNet	0.450*^‡†^ [0.326, 0.579]	0.867 [0.793, 0.932]
CheXMax	0.900^^‡†^ [0.773, 1.00]	0.767*^‡†^ [0.672, 0.857]
Augmented HITL model (combined swarm and CheXMax)	0.875*^‡†^ [0.783, 0.956]	0.933^ϕ^ [0.877, 0.983]

*N/A* not applicable

^^^Indicates a statistically significant difference (*p* < 0.01) compared to group A swarm interpolation

*Indicates a statistically significant difference (*p* < 0.05) compared to group A swarm interpolation

^¶^Indicates a statistically significant difference (*p* < 0.01) compared to group B swarm interpolation

^‡^Indicates a statistically significant difference (*p* < 0.05) compared to group B swarm interpolation

^†^Indicates a statistically significant difference (*p* < 0.01) compared to combined swarm interpolation

^Ø^Indicates a statistically significant difference (*p* < 0^.^05) compared to combined swarm interpolation

^ϕ^Indicates a statistically significant difference (*p* < 0.01) compared to CheXMax

^Ψ^Indicates a statistically significant difference (*p* < 0.05) compared to CheXMax

^a^Data reported as mean [95% confidence interval] as applicable, unless otherwise specified

The sensitivity and specificity of each of the diagnostic methods is compared in Table 2. In terms of the sensitivity of each diagnostic method, the swarm outperforms CheXNet in all groups (a combined group sensitivity of 0.700 [0.578, 0.814] versus CheXNet’s 0.450 [0.326, 0.579], *p* < 0.01), while CheXMax and the combined model outperform the swarm in all groups, with sensitivities of 0.900 [0.773, 1.00] (*p* < 0.05) and 0.875 [0.783, 0.956] (*p* < 0.05), respectively. In terms of the specificity of each diagnostic method, the swarm outperforms CheXMax, with a specificity of 0.933 [0.855, 0.968] compared to CheXMax’s specificity of 0.767 [0.672, 0.857] (*p* < 0.01). The Augmented HITL model obtains a specificity of 0.933 [0.877, 0.983], the same as the swarm. Interestingly, the human diagnostic methods all show a lower sensitivity than specificity, while CheXMax shows a higher sensitivity than specificity. The swarm shows the highest specificity of any of the diagnostic methods, while CheXMax shows the highest sensitivity of any of the diagnostic methods.

Due to the differences in sensitivity and specificity between the swarm and CheXMax, it is clear that these two diagnostic methods have different strengths and advantages when diagnosing pneumonia: CheXMax has a higher sensitivity, so this machine-learning model is more precise at detecting pneumonia if it does exist, whereas the swarm has a higher specificity, so this human-driven model is more precise at detecting when pneumonia does not exist in an image. An augmentation model was created to determine whether these two strengths could be combined into a single model to achieve higher accuracy as compared to either model alone. CheXMax was used first to diagnose the probability of pneumonia across all 50 cases. Cases in which CheXMax yielded a low-confidence diagnosis were then passed on to swarm. Low-confidence was defined as a CheXMax probability between 2.5% and 5.5%, resulting in 20 cases passed on to swarm for final prediction of pneumonia. This selection was chosen as a fair low-confidence band around the average CheXMax prediction (*p* = 4.008%) and yielded 20 cases on which to swarm: 13 negative predictions and 7 positive predictions. Each swarm’s probabilistic diagnoses on these 20 cases were then used as the augmented model’s final diagnoses, in place of the ML system’s low-confidence diagnoses. All other cases remained diagnosed exclusively by the ML system.

This augmented combined system achieved a statistically higher diagnostic performance than either CheXNet or CheXMax and combined swarm alone using two of the performance metrics (e.g. accuracy 92% vs. 82% and 84%; F1 score of 0.89 vs. 0.80 and 0.78, respectively; *p* < 0.01) (Table 1). The augmented model system achieves the lowest diagnostic error rate, in terms of number of diagnoses incorrect, compared to any of the other examined diagnostic methods (error rates of 9% augmented model; 16% combined swarm; 18% CheXMax; 24% individuals). Moreover, the augmented system achieves near-best performance in both sensitivity and specificity, with a sensitivity of 0.875 [0.783, 0.956] and a specificity of 0.933 [0.877, 0.983]. It appears that this augmented model is therefore able to combine the best aspects of both the machine-learning system (CheXMax), which has high sensitivity, and the swarm, which has high specificity.

**Fig. 2:**
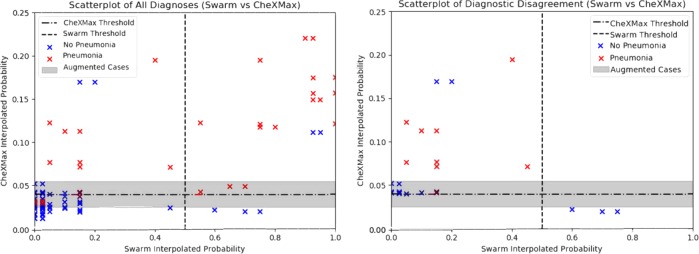
Scatterplot of swarm vs. CheXMax probabilistic diagnoses, with cases colored by ground truth. The scatterplots show that CheXMax and human swarms assign very different probabilities to each case (left image). The gray “Augmented Cases” range shows cases that were sent from CheXMax to the Swarm for augmentation. CheXMax has a high incidence of True Positives (blue-colored cases below the horizontal CheXMax Threshold line), but when the CheXMax gives a weak positive diagnosis (between 0.04008 and 0.055 on the *y*-axis), it is often incorrect (11 out of 15 cases correct, or an accuracy of 73%). Using a human swarm to re-classify these weak positive cases results in correctly labeling 14 out of 15 of the cases—an accuracy improvement of 20%. The cases on which the two diagnostic methods disagreed are more clearly visualized in the scatterplot of diagnostic disagreement (right image).

To better visualize how the HITL augmentation process changed the probabilistic diagnoses of CheXMax and why these changes resulted in a more accurate system, a scatterplot of probabilistic diagnoses is shown in Fig. 2. Over all 100 of the evaluated cases, the swarm and CheXMax disagreed on a total of 24, of which the vast majority (21, or 87.5%) were cases where the swarm gave a negative diagnosis, but CheXMax gave a positive diagnosis. Over all 24 cases where the Swarm and CheXMax disagreed, the swarm was correct on 12 diagnoses, while CheXMax was correct on the other 12 diagnoses.

**Fig. 3:**
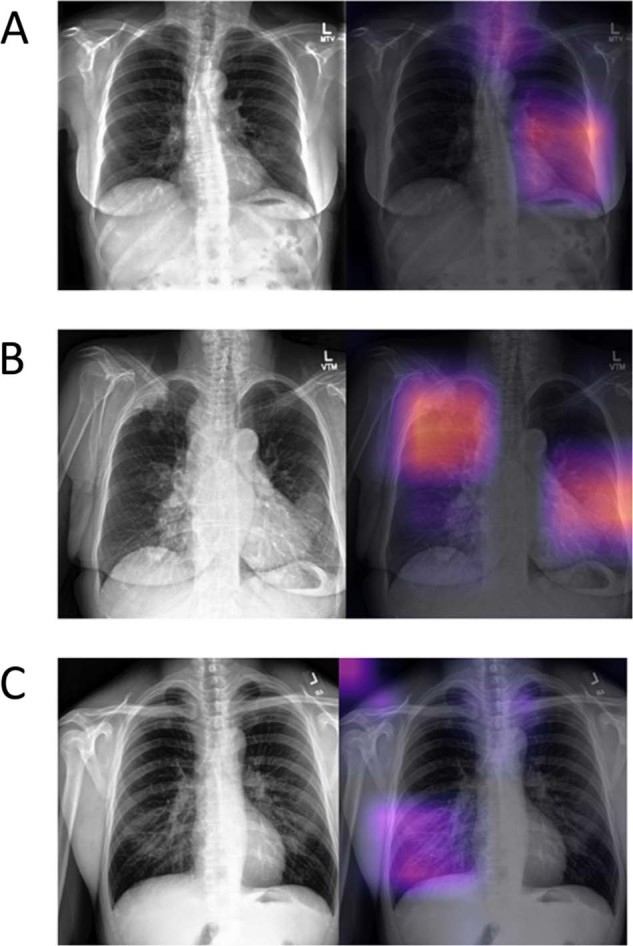
Case examples. Each of the three rows **a**–**c** represent three different patients. Grayscale image is on the left with the corresponding class activation map to its right. The top row example **a** includes a patient with pneumonia in the left lung, correctly predicted by CheXMax but incorrectly by swarm. The middle row **b** is an example of a patient with metastatic disease but without pneumonia, correctly predicted by swarm and incorrectly by CheXMax. The bottom row **c** is an example of an augmented case, where CheXMax provided a low confidence positive prediction (*p* = 0.41) but was correctly predicted as negative by swarm.

The gray band across each image represents the range of cases that CheXMax diagnosed with low confidence (probabilistic diagnosis between 0.025 and 0.055), and subsequently sent to the swarm for a second opinion. These 20 cases were each evaluated by both Group A and Group B, for a total of 40 diagnoses generated by the swarms in the augmented system. Of these 40 diagnoses, 29 agreed with CheXMax’s original evaluation: three cases where both diagnostic methods gave a positive reading, with 100% accuracy, and 26 cases where both methods gave a negative reading, with 84.6% accuracy. Only 11 diagnoses disagreed with CheXMax’s original evaluation, all of which were low-confidence positive diagnoses by CheXMax, and high confidence negative diagnoses by swarm (less than a 20% probability of pneumonia). Of these 11 cases, the swarm correctly changed CheXMax’s original diagnosis 10 out of 11 times (91%). Figure 3 provides examples of correct diagnosis by CheXMax over swarm, and vice-versa, as well as an augmented case with correct diagnosis changed by swarm from CheXMax.

Because the boost in performance of the augmented model depends on the selection of cases (i.e. how “low confidence” is defined) sent to the swarm, a sensitivity analysis was performed to determine the impact of the quantity of cases selected to be sent to the swarm on the accuracy of the augmented model. In this sensitivity analysis, cases are selected based on their distance to the discrimination threshold (4.008%) of CheXMax, starting with the lowest-confidence cases (those closest to the discrimination threshold). An equal number of positive and negative cases are selected to be diagnosed with Swarm at each cutoff, where the number of cases selected ranges from 0 to 50 (0–100% of the data). The number of cases correctly diagnosed by the augmented system is calculated for each cutoff.

**Fig. 4:**
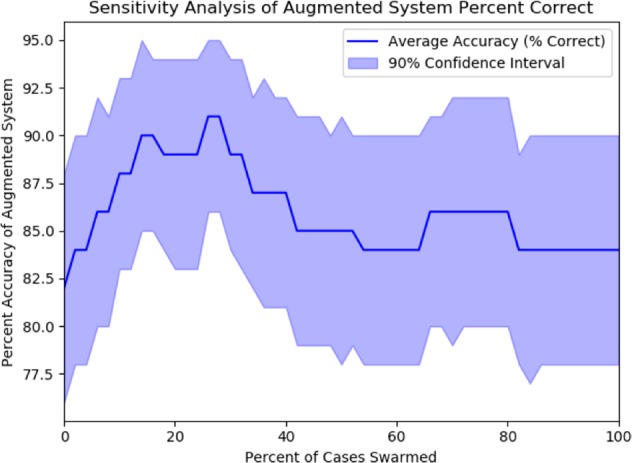
Sensitivity analysis of augmented model accuracy. The shape of the average accuracy line shows a consistent increase in the accuracy of the augmented model when the 0–14% lowest-confidence cases are sent to the swarm, from 82% correct of CheXMax (sending 0% of cases) to 90% correct when sending the 14% of lowest-confidence positive and negative cases to the swarm. The model performs similarly when 16–32% of cases are sent to the swarm, achieving between 88% and 92% accuracy across this sensitivity range. If more than 32% of cases are sent to the swarm, the accuracy of the system decreases, until the limit of sending all diagnoses to the swarm is reached (100% of cases swarmed), where the accuracy returns to the swarm score of 84%.

**Fig. 5:**
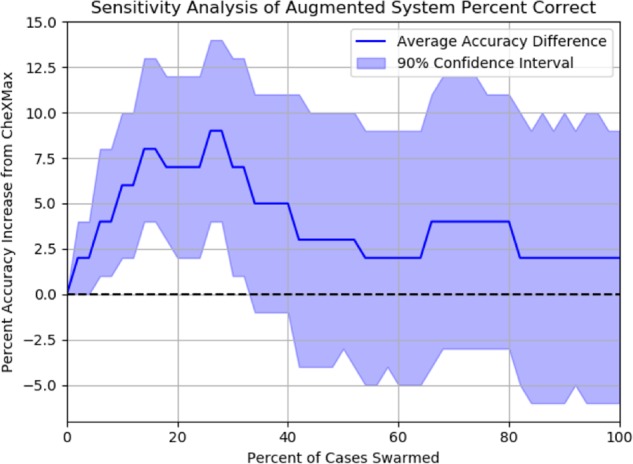
Sensitivity analysis of accuracy increase relative to CheXMax. Sensitivity analysis shows a band between 6% and 34%, where the 90% confidence interval is only ever >0%. This indicates that when sending between 6% and 34% of the lowest-confidence cases to the swarm using this method, there is high confidence that the augmented model would diagnose the cases more accurately than the CheXMax alone. If the range is limited between 14% and 28%, the average improvement in accuracy is 7.75% correct.

**Fig. 6:**
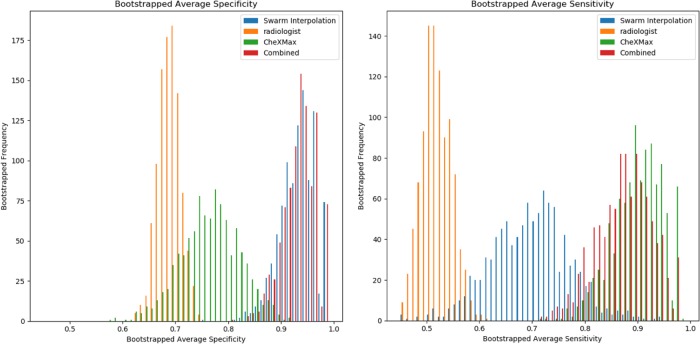
Bootstrapped average specificity and sensitivity of aggregate diagnostic methods. The bootstrapped specificity histograms show that the swarms in the combined group (blue bars) outperform CheXMax (green bars) in terms of specificity (left image), but CheXMax outperforms the swarms in terms of sensitivity (right image). The HITL Combined model combines the best of both the CheXMax and swarm diagnostic methods, by attaining swarm-level specificity and CheXMax-level sensitivity.

A bootstrapping analysis with 1000 bootstraps is used to find the 90% confidence interval of accuracy for each cutoff by randomly re-sampling a full set of cases from the observed population 1000 times and calculating the observed accuracy for each resampled set of cases. The average accuracy increases of this augmented system relative to CheXMax and 90% confidence interval of this accuracy increase are shown in Figs. 4 and 5. This sensitivity analysis suggests that the success of the augmented model is only slightly sensitive to the choice of threshold at which a case is deemed “low-confidence”. Regardless of the choice of threshold, the average accuracy of the augmented system is greater than or equal to that of the ML system (Fig. 6). When the proportion of cases sent to the swarm is between 6% and 32%, however, the augmented system diagnoses more cases correctly than the ML system alone (*p* < 0.05), indicating that the augmented model outperforms CheXMax across a wide range of “low confidence” thresholds.

## Discussion

Our study shows that, using a test set of 50 chest radiographs with strong ground truth using clinical outcomes, highest diagnostic performance can be achieved with HITL AI when radiologists and AI technologies work together. We combined a novel real-time interactive platform that utilizes the biological concept of swarm intelligence with a deep-learning model and found the maximum diagnostic performance that neither alone was able to achieve.

Using a swarm platform, we found that the diagnostic performance was higher than individual radiologist performance in diagnosing pneumonia. Moreover, when results of both swarm sessions were combined, swarm-based diagnoses outperformed crowd-based majority vote. This has important implications as many studies involving deep-learning models often use either individual expert, consensus, or majority vote to provide ground truth labels for validation and test sets when stronger metrics, such as pathology results, are unavailable or not applicable.^4,15,24,25^ Results from our study shows that swarm-based diagnoses outperforms crowd-based diagnoses, and thus may represent a novel means for generating image labels that provide more accurate ground truth than conventional consensus labeling methods for training datasets for deep-learning algorithms. Moreover, some centers may not readily have access to experts, and labeling images through swarm sessions may allow such centers to achieve expert level labels.

It has been well-established that crowds of people can outperform individuals and achieve estimates close to the true value that would otherwise not be possible with individual estimates, a concept known as “wisdom of crowd effect”.^26–30^ In fact, the use of this effect specifically for medical decision making has also been described.^31–34^ However, this effect is a statistical aggregation of individual estimates. Traditionally, its power is shown through votes, conducting polls, or collecting surveys such that the input from each individual member is captured in isolation (or near isolation) and then combined with the data collected from other members to then pass through a post-hoc statistical processing. Thus, the dynamics of a real-time collaboration and negotiation are void within these types of “crowds.” Studies have also shown that wisdom of crowd effect could be undermined by social influence, and that the crowd-based decision can be biased resulting in tending away from higher accuracy compared to the individual.^28,35,36^ This is, however, dependent on how the communication network for information exchange is structured.^37^

Modeled after complex decision processes used by swarming honeybees, the real-time algorithms that connect users of the Swarm platform enable human groups to work together to integrate noisy and incomplete information, weigh competing alternatives, and converge on unified decisions in real-time synchrony. In this way, the swarm-based technology utilized in this study enabled networked groups of radiologists to outperform individual radiologists, groups of radiologists taking a traditional vote, and the CheXNet deep-learning system when diagnosing pneumonia on chest radiographs.

Similar to the human experiment in which we aimed to harness the maximum diagnostic potential, we retrained CheXNet,^38^ which was originally trained on a publicly available NIH dataset,^39^ on a recently released large dataset of chest radiographs with radiologist level validation and test sets.^5^ This newly trained deep-learning algorithm, CheXMax, outperformed the average radiologist for the detection of pneumonia (e.g. 82% vs. 76% accuracy, respectively). Moreover, this newly trained algorithm outperformed the swarm-based method when using the AUC metric (the standard measure of diagnostic classification accuracy) and underperformed the swarm-based method when using mean absolute error and Brier score (the standard measures of probabilistic accuracy) (Table 1). Since the AUC metric measures the success of ordering the cases from least to most likely to contain pneumonia, while the Brier score and mean absolute error scores measure the probabilistic accuracy of the diagnoses—e.g. whether a diagnosis of a 10% chance of pneumonia actually contained pneumonia only 10% of the time—this result suggests that the CheXMax is better at ordering the cases from least to most likely to contain pneumonia, while the swarm is better at assessing the probabilistic likelihood of pneumonia in a specific case.

As deep-learning models continue to improve though larger and higher quality dataset for training, as we found with retraining CheXNet,^5^ an unanswered question remains as to what the exact scenario of implementation within clinical workflow will be. Advantages of deep-learning algorithms include rapidity in diagnosis proving to be useful as a triage tool. Disadvantages include biases introduced by training dataset and inability to contextualize to clinical context.^8,11,12,40^ Thus, many have advocated that a clinical workflow model in which healthcare workers leverage AI might yield the greatest benefit to patients.^40,41^ To that extent, few studies have shown superior performance of human augmented by AI compared to either human or machine alone.^15,16^ In our study, we showed the ability of a deep-learning algorithm in CheXMax to provide rapid confident diagnoses for pneumonia for over a half of the cases in the test set. Low-confidence cases were then passed on to the human through swarm to yield the final diagnosis and the combined HITL model resulted in higher diagnostic accuracy than either radiologists or AI models alone. The clinical significance of this could imply that, in a landscape of increasing clinical volumes, complexity of cases, and medical record documentation, physicians could leverage deep learning to improve operational efficiency; deep-learning algorithms could provide automated rapid diagnosis for high confident cases as a triage tool so that physicians could spend less time on high confidence cases evaluated by an AI model and more time on relatively complex cases. In such HITL scenarios, active learning could be provided to AI algorithms through feedback from radiologists in the form of additional training data, which the model did not initially provide a confident diagnosis. Further studies are needed to determine the optimal workflow and implementation of deep-learning algorithms in the healthcare setting.

Several limitations of our study merit consideration, including those that are attributed to a retrospective design; despite having a strong clinical reference standard, we utilized a very small test set of 50 cases which, though achieving the best available clinical ground truth, nonetheless, it is possible that patients who were included may have had other pathologies that were clinically treated as pneumonia in routine clinical care, which may have confounded the diagnosis had the follow-up period been prospectively designed or more invasive testing been performed (i.e. sputum cultures, bronchoscopy, direct sampling), which may have altered the results of the study. This size was chosen as to practically perform swarm sessions with synchronous groups of radiologists in a timely manner, and judging by the statistical analysis in this pilot it is possible that larger sample sizes would have observed a similar trend. While we studied the improved diagnostic accuracy of combining the capabilities of CheXMax with swarm, we did not perform a simulation experiment where CheXMax results are given real-time during the swarm sessions. The cutoff for the model decisions were selected by maximizing Youden’s *J* on the validation set and this low probability leads to a good tradeoff between sensitivity and specificity for this task. Finally, one could argue that such a platform as swarm may not be practically necessary for decisions and tasks as relatively simple as diagnosing pneumonia on chest radiographs.

In conclusion, we demonstrated increased diagnostic performance of radiologists in diagnosing pneumonia on chest radiographs through swarm intelligence using a novel platform, superior performance of a deep-learning algorithm trained on higher quality data compared to individual radiologists and achieved the maximum diagnostic accuracy using an HITL AI approach through an augmented model combining both radiologists (through swarm) and AI (using a deep-learning model). Although we focused on one common medical-imaging task, future work could assess the feasibility and application for other various medical diagnostic tasks and decision making.

## Methods

This Health Insurance Portability and Accountability Act-compliant study was approved by the Institutional Review Board of Stanford University, and a waiver of informed consent was obtained.

### Chest radiograph pneumonia dataset

We retrospectively searched our electronic medical record database and picture and archiving communications system (PACS) for patients who underwent chest radiographs (anterioposterior or posterioanterior) in the emergency room or in the outpatient clinic setting over a 2-year period between 2015 and 2017. The search yielded an initial target population of 7826 unique patients with 11,127 chest radiographs. Patients were eligible for inclusion in the study if they presented with clinical signs and symptoms concerning for pneumonia, such as fever, cough, shortness of breath, elevated white blood cell count, crackles on physical examination, etc.^42,43^ Subjects were excluded from the study if: (a) the clinical reference standard was inadequate (see below) or (b) an inadequate examination due to a suboptimal technique or incomplete imaging data available. The first consecutive 50 unique patients who met the aforementioned eligibility were included in the final population. A test set size cutoff of 50 chest radiographs was used in order to practically perform the human reader evaluation in a timely fashion and so as not to introduce reader fatigue that might occur with larger datasets. The final retrospective cohort was comprised of 27 males and 23 females (mean age ± standard deviation, 62.1 ± 21.0 years; range, 19–100 years) with a test set of 50 frontal chest radiographs.

### Clinical reference standard

Only those patients and their frontal chest radiographs were included, if they are presented with aforementioned signs and symptoms and clinical concern for pneumonia. An image was labeled negative if all of the following criteria were met: (a) chest radiograph was interpreted as negative for pneumonia by a board-certified diagnostic radiologist at the time of examination; (b) a follow-up chest computed tomography (CT) within 1 day after the index chest radiograph confirmed lack of pneumonia on imaging; (c) the patient was not administered antibiotics. An image was labeled positive for pneumonia if all of the following criteria were met: (a) chest radiograph was interpreted as positive for pneumonia by a board-certified diagnostic radiologist at the time of examination; (b) patient was treated with antibiotics; (c) a follow-up chest CT or chest radiograph within 7 days after treatment showed interval improvement or resolution of pneumonia; (d) patient showed clinical signs of improvement after treatment on follow-up visit. Using this reference standard, the test set contained a class balance of 30 negative and 20 positive exams for pneumonia.

### Deep-learning models and architectures

Two previously developed and described state-of-the-art convolutional neural networks for chest radiographs were used.^4,5^ First, a 121-layer dense convolutional neural network (DenseNet), CheXNet, was used on the 50 test cases. This model was trained using the publicly available dataset released by Wang et al.^39^ CheXNet was previously tested on 14 different chest radiograph pathologies, including pneumonia, and outperformed a group of board-certified diagnostic radiologists^5^ as well as previous models^39,44^ using the same dataset. Though large datasets, such as the one released by Wang et al. ^39^ have allowed progress in deep-learning automation of chest radiographs, those efforts can only achieve a certain advancement before reaching a plateau. This is due to the fact that large well-labeled datasets with strong clinical reference standards are needed. Publicly available large datasets are often limited as the labels are derived from automatic labelers that extract information from existing radiology reports.^39^ Additionally, these labelers cannot account for uncertainty that may be conveyed in free text radiology reports. Thus, the advantages of access to available large datasets can come at a cost of weak labels. A recent large dataset of chest radiographs was released that addresses these limitations with labels that account for uncertainty and has strong reference standards with radiologist labeled validation and test sets.^5^ Using the this recently released database, we retrained CheXNet model (the newly trained model referred to as CheXMax), hypothesizing that the improved training dataset would boost the diagnostic potential of this deep-learning algorithm for chest radiographs. The test set of 50 chest radiographs were evaluated with CheXMax and probabilities of pneumonia for each exam were derived.

### Radiologists

A total of 13 board certified diagnostic radiologists (average years of experience: 7.8 years; range 1–23 years) across two major busy tertiary care centers (Stanford University and Duke University) participated in this study. The 13 radiologists were arbitrarily divided into two groups (group A—7 radiologists, average(range) of experience: 6.6 (1–11); group B—6 radiologists, average(range) of experience: 9.2 (1–23)) based on their availability. Each group participated in a 2-h session (see the “Swarm sessions” section) to evaluate a test set of 50 chest radiographs, first individually and then as a swarm.

### Swarm platform and model architecture

**Fig. 7:**
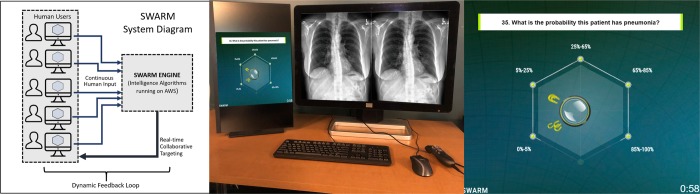
Swarm platform. A system diagram (left image) of the Swarm platform shows the connection of networked human users. A Swarm engine algorithm received continuous input from the humans as they are making their decision and provides real-time collaborative feedback back to the humans to create a dynamic feedback loop. Swarm Platform positioned next to a second screen for viewing radiograph (middle image). A snapshot (right image) of the real-time swarm of six radiologists (group B) shows small magnets controlled by radiologists pulling on the circular puck in the process of collectively converging towards a probability of pneumonia. To view a video of the above question being answered in the Swarm platform, visit the following link: https://unanimous.ai/wp-content/uploads/2019/05/Radiology-Swarm.gif.

In order to assess both individual diagnostic performance and maximal collective human diagnostic performance, we employed a novel real-time collaborative software platform called Swarm that has been assessed in a variety of prior studies and has been shown to amplify the combined intelligence of networked human groups.^23,45–47^ While traditional systems that harness the intelligence of groups collect data from participants in isolation, usually through an online survey, and then combine the input statistically to determine the group response, the Swarm platform enables participants to work together in real-time, converging on a group decision as a unified system that employs biological principle of Swarm Intelligence. This is achieved using a unique system architecture that includes a central processing engine that runs swarming algorithms on a cloud-based server (Fig. 7a). The processing engine is connected over the internet to a set of remote workstations used by the human participants (Fig. 7b). Each workstation runs a client application that provides a unique graphical interface for capturing real-time behavioral input from participants and for providing real-time feedback generated by the processing engine.

The processing engine employs algorithms modeled on the decision-making process of honeybee swarms. The underlying algorithms enable networked groups to work together in parallel to (a) integrate noisy and incomplete information, (b) weigh competing alternatives, and (c) converge in synchrony on optimized decision, all while allowing participants to react to the collective impact they are having on the changing system in real-time, thereby closing a feedback loop around the whole group.^21^ To use this platform, distributed groups of participants (in this case radiologists) log on to a central server from their own individual workstations and are simultaneously asked a series of questions to be answered together as a swarm. In this study, each question in the series involved assessing the probability of a patient having pneumonia based upon a displayed chest radiograph.

To answer each question, the participants collaboratively move a graphical pointer represented as a glass puck (Fig. 7c). An answer is reached when the group moves the puck from the center of the screen to a target associated with one of the available answer options. In this study, the displayed question was “*What is the probability this patient has pneumonia?”* and the answer options were five percentage ranges that the participants could choose among. The ranges were (0–5%), (5–25%), (25–65%), (65–85%), and (85–100%).

To influence the motion of the puck, each participant controls a graphical magnet using their mouse or touchscreen. The magnet enables each participant to express their intent upon the collaborative system by pulling the graphical puck in the direction they believe it should go. It is important to note that these user inputs are not discrete votes, but continuous streams of vectors provided simultaneously by the full set of participants, enabling the group to collectively pull on the system in opposing and/or supporting directions until they converge, moving the puck to one solution they can best agree upon. It is also important to note that the impact that each user has on the motion of the puck is determined by the swarm algorithms at every time step. The algorithms evaluate the relative conviction that each participant has at each moment based on their behaviors over time (i.e. how their magnets move as compared to each of the other participants). In this way, the software enables real-time control system such that (i) the participants provide behavioral input at every time-step, (ii) the swarming algorithms determine how the graphical pointer should move based on the behavioral input, (iii) the participants to react to the updated motion of the pointer, updating their behaviors in real-time, and (iv) the swarming algorithms react to the updated behaviors, thereby creating a real-time, closed-loop feedback system. This process repeats in real-time until the participants converge on a final answer by positioning the pointer upon one of the five targets.

Using this method, the distributed group of users quickly converge on solutions, each answer being generated in under 60 s. After the solutions is reached, the behavioral data is fed into an interpolation algorithm which computes a refined probability as to the likelihood that patient associated with the displayed radiograph is positive for pneumonia. This interpolation is performed because the group of participants were provided a simple set of five options to choose from, each representing a wide range of probabilities. By interpolating the behavioral data captured while the group guided the puck to the target, a refined probability value can be computed with a high degree of precision.

### Swarm sessions

Two groups (A and B) of radiologists participated in two separate swarm sessions, split randomly based on the availability of each radiologist to participate on a given date. Each session diagnosed 50 cases. For each case, participants were first asked to view a DICOM image of a frontal chest radiograph using their own independent workstation with a DICOM viewer of their preference. Individual assessments of the probability of pneumonia within this image were made through an online questionnaire using the swarm platform. These individual assessments were not revealed to other participants. Individuals were not given a time limit for the completion of the online questionnaire, and never took more than 1 min to review each image and complete the questionnaire. Subsequently, the group worked together as a real-time swarm, converging on a probabilistic diagnosis as to the likelihood that the patient has pneumonia using the aforementioned magnets to move the puck. The radiologists had no direct communication during the swarm and were anonymous to one another. The diagnosis was arrived at through a two-step process in which the swarm first converged on a coarse range of probabilities and then converged on a refined value within the chosen range. The full process of deliberation for each case, as moderated by the real-time swarm artificial intelligence algorithm, generally took between 15 and 60 s. No swarm failed to reach an answer within 60 s. Each swarm session took 2 h to complete the entire test set.

### Statistical analysis

Probabilities produced by *CheXNet and CheXMax* were converted to binary prediction using a discrimination threshold (*p* = 50.0% for CheXNet and *p* = 4.006% for CheXMax). Similarly, for the human assessments of chest radiographs prior to the swarm-based decision, probabilities of pneumonia were converted to binary prediction using a 50% threshold—any diagnoses >50% probability were labeled as “pneumonia predicted”. This was performed for individual radiologist diagnoses, the average of all radiologist diagnoses for a single image, as well as by a crowd-based majority vote. For the swarm session, results of the two separate sessions were analyzed separately as well as together. The final probability selected by the swarm was further refined through using underlying data generated during the convergence process. This was done using a weighted averaging process referred to as squared impulse interpolation or swarm interpolation. This process, as outlined in equations below, calculates a weighted average of the probabilities in the swarm using the squared net “pull” towards each answer as weights. The pull is represented as the force (*F*) imparted by members of the swarm and the weight for each answer *w*_*i*_ is calculated as the squared impulse towards that answer (Eq. (1)). The weighted average over the answer choice values *v*_*i*_ is then computed (Eq. (2)). The answer choice values *v*_*i*_ are taken as the midpoint of each bin. For example, the bin “0–5%” has a midpoint *v*_*i*_ of 2.5%.

1 $$w_i = \frac{{F(i)^2}}{{\mathop {\sum}\nolimits_{a \in {\mathrm {Answers}}} F(a)^2}}$$

2 $${\mathrm {Refined}}\,{\mathrm {probabilistic}}\,{\mathrm {diagnosis}}\,{\sum} {w_iv_i}$$

**Fig. 8:**
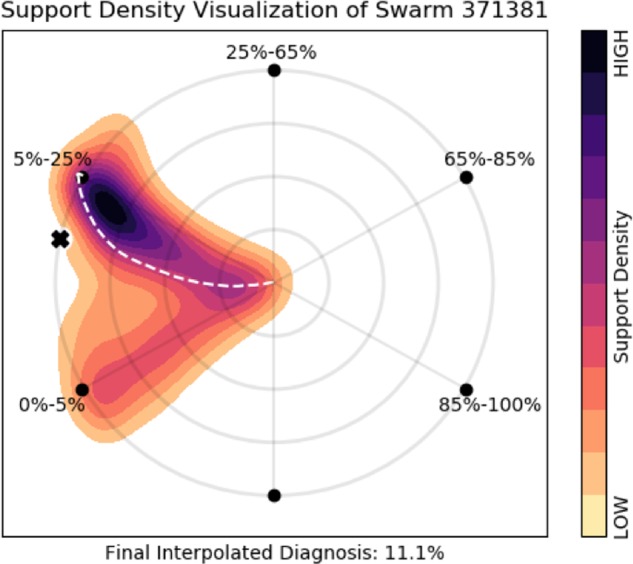
Support density visualization. In this support density visualization corresponding to the swarm in Fig. 1, the puck’s trajectory is shown as a white dotted line, and the distribution of force over the hex is plotted as a Gaussian kernel density heatmap. Notice that this swarm was split between the “5–25%” and “0–5%” bins, and more force was directed towards the 5–25%. This aggregate behavior is reflected in the swarm’s interpolated diagnosis of 11.1%.

This process can be visualized by plotting the net vector force of each radiologist over the course of the swarm, as shown in Fig. 8.

Final diagnostic performance was compared between radiologists (average performance of individual radiologists, averaging individual diagnoses on an image within a group to calculate the group’s average probabilistic diagnosis, and taking a vote of individual radiologist diagnoses to label the image in a binary manner), the AI models, and diagnosis by swarm. Five different diagnostic performance metrics were used to make the comparisons: (a) percent correct, (b) mean absolute error, (c) Brier score, (d) AUC, and (e) F1 score.

### Reporting summary

Further information on research design is available in the Nature Research Reporting Summary linked to this article.

## Supplementary information

Reporting Summary Checklist

## Data Availability

Data are available on request due to privacy or other restrictions. The data that support the findings of this study are available on request from the corresponding author (B.N.P.). The data are not publicly available due to privacy information embedded directly within the data.

## Code availability

Restrictions apply regarding the release of source code used in this study.
