# Author Correction: Human–machine partnership with artificial intelligence for chest radiograph diagnosis

**DOI:** 10.1038/s41746-019-0198-6

**Published:** 2019-12-10

**Authors:** Bhavik N. Patel, Louis Rosenberg, Gregg Willcox, David Baltaxe, Mimi Lyons, Jeremy Irvin, Pranav Rajpurkar, Timothy Amrhein, Rajan Gupta, Safwan Halabi, Curtis Langlotz, Edward Lo, Joseph Mammarappallil, A. J. Mariano, Geoffrey Riley, Jayne Seekins, Luyao Shen, Evan Zucker, Matthew P. Lungren

**Affiliations:** 1 Department of Radiology, Stanford University School of Medicine, 300 Pasteur Dr., H1307, Stanford, CA 94305 USA; 2 Unanimous AI, 2443 Fillmore Street #116, San Francisco, CA 94115-1814 USA; 3 Department of Computer Science, Stanford University School of Medicine, 353 Serra Mall (Gates Building), Stanford, CA 94305 USA; 4 Department of Radiology, Duke University Medical Center, Box 3808 Erwin Rd, Durham, NC 27710 USA

**Keywords:** Radiography, Computer science

**Correction to:** *npj Digital Medicines* 10.1038/s41746-019-0189-7, published online 18 November 2019

The original version of the published Article omitted of the last Author’s middle initial. “Matthew Lungren” has been changed to “Matthew P. Lungren”. This has been corrected in the HTML and PDF version of the Article.

